# Quasicontinuum Simulation of the Effect of Lotus-Type Nanocavity on the Onset Plasticity of Single Crystal Al during Nanoindentation

**DOI:** 10.3390/nano8100778

**Published:** 2018-09-30

**Authors:** Jianfeng Jin, Peijun Yang, Jingyi Cao, Shaojie Li, Qing Peng

**Affiliations:** 1 Key Laboratory for Anisotropy and Texture of Materials, Ministry of Education, School of Materials Science and Engineering, Northeastern University, Shenyang 110819, China; yangpj_yangcx@163.com (P.Y.); neu_caojy@163.com (J.C.); lisj_neu@163.com (S.L.); 2 Nuclear Engineering and Radiological Sciences, University of Michigan, Ann Arbor, MI 48109, USA

**Keywords:** quasicontinuum method, onset plasticity, nanosized cavity, geometry effect, nanoindentation

## Abstract

Stress concentration around nanosized defects such as cavities always leads to plastic deformation and failure of solids. We investigate the effects of depth, size, and shape of a lotus-type nanocavity on onset plasticity of single crystal Al during nanoindentation on a (001) surface using a quasicontinuum method. The results show that the presence of a nanocavity can greatly affect the contact stiffness (*S_c_*) and yield stress (*σ_y_*) of the matrix during nanoindentation. For a circular cavity, the *S_c_* and *σ_y_* gradually increase with the cavity depth. A critical depth can be identified, over which the *S_c_* and *σ_y_* are insensitive to the cavity depth and it is firstly observed that the nucleated dislocations extend into the matrix and form a *y*-shaped structure. Moreover, the critical depth varies approximately linearly with the indenter size, regarding the same cavity. The *S_c_* almost linearly decreases with the cavity diameter, while the *σ_y_* is slightly affected. For an ellipsoidal cavity, the *S_c_* and *σ_y_* increase with the aspect ratio (*AR*), while they are less affected when the *AR* is over 1. Our results shed light in the mechanical behavior of metals with cavities and could also be helpful in designing porous materials and structures.

## 1. Introduction

As a kind of defect, cavities change materials’ properties and result in an inhomogeneous distribution of the internal stress field. It is crucial to understand the effects of cavities on mechanical properties of materials [1]. This knowledge is important in the design of porous nanoscale materials, such as nano-drilled materials [2,3], and lotus-type porous metals [4,5] that exhibit good sound absorption and vibration-damping properties for the applications of heat sink, golf putter, and biomaterials. Many researchers have attempted to study the effect of cavities on the mechanical behavior of materials at scales from the microscopic to macroscopic, using both experiments [6,7,8,9,10] and simulations [11,12]. These studies show that nucleation, growth, and coalescence of cavities at grain boundaries, inclusions, or inter granular interfaces are the main causes of fracture in plastic materials [13,14,15,16]. Tane et al. [17] investigated the formation mechanism of a plateau stress region in the stress-strain curves during the dynamic compression of porous iron, which resulted in an excellent property of absorption of high-impact energy, and was caused by the formation of deformation bands, owing to the interaction between oriented cylindrical pores, and deformation twins.

Nanoindentation technology has been widely used to evaluate the mechanical properties of bulk materials [18,19] and thin films [20]. For example, Ehtemam-Haghighi et al. [18] used nanoindentation to study the hardness, elastic modulus, and wear resistance of Ti based alloys with Fe and Ta additions at a micro-scale, in which dislocation density and morphological distribution played important roles for mechanical response. However, when the sample or indentation size decreases, the mechanical response is quite different [21,22]. In Horstemeyer et al. [21], they showed the stress-strain responses at four different size scales (i.e., macroscale, microscale, submicro scale, and nanoscale). At nanoscale, nanoindentation is used to study onset plasticity [23], which is crucial in applications such as nanoelectromechanical systems (NEMS) [24]. For an ultrasmall sample, which is nearly a dislocation-free material, onset plasticity is dominated by dislocation nucleation rather than the morphological distribution or dislocation density [23]. For example, Minor et al. [25] proposed an in-situ view of the onset plasticity in a submicrometre grain of Al during nanoindentation and found that the corresponding shear stress was similar to the theoretical shear strength. Tsuru et al. [26] investigated the elastic and plastic deformation in Al and Cu single crystals during nanoindentation using the molecular dynamic (MD) method, where the results showed that the collective dislocation emission and the formation of surface steps triggered a load drop for the onset of plasticity.

On the basis of the continuous surface elasticity, Li et al. [27] studied the effect of the surface on the stress concentration around a spherical cavity in anisotropic elastic medium using a continuum model. Their results indicated that the surface effect led to a dependence of the stress concentration factor on the cavity size. Ou et al. [28] analyzed the effect of residual surface tension on the elastic stress field around spherical cavities in a continuum model; their results indicated that the elastic field near the spherical cavity strongly depended on the surface tension and on the shape and size of the cavities, especially in the case of nanosized cavities. The stress concentration caused by such nanosized cavities during deformation can greatly affect mechanical response, which was strongly related to dislocation behaviors [29]. However, the traditional continuum model cannot treat atomic defect structures such as dislocation cores accurately. Using both MD and finite element methods, Wu et al. [30] studied the effects of the volume fraction and shape of the nanocavities on the elastic properties of nanocrystalline materials. Using MD simulation, Yang et al. [31] found that the modulus linearly depended on the volume fraction of the round-shaped cavity and nonlinearly on its shape during the application of uniaxial tension to nanocrystalline sheets. Moreover, it was observed that the interaction between the cavities and the interface strongly affected the elastic and plasticity responses of materials at the nanoscale, although studies of this interaction would require a larger spatial simulation cell.

To overcome the spatial limitation of the MD method, Tadmor and Miller developed the quasicontinuum (QC) model by seamlessly coupling continuum and atomistic simulation [32]; their approach enabled an atomic simulation on a larger spatial scale without a degradation of the accuracy. Later, Peng et al. revised the QC model with orbital-free density-functional theory [33] and used it to study the mechanical response of an Al thin film in the presence of Mg impurities during nanoindentation [34]. Jin et al. [35] used the QC method to study the onset plasticity of the Al (001) surface during nanoindentation and found that the onset plasticity was closely related to dislocation nucleation and twinning formation. Recently, Yu et al. [36] used the QC method to investigate the effects of the shape and position of the cavity on mechanical properties of the Cu film during nanoindentation on a (110) surface. The results showed that the onset plasticity was substantially diminished by the corresponding dislocation nucleation from the inner side of the cavities. Using the QC model, Péron-Lührs et al. [37] investigated the effect of nanosized cavities at grain boundaries on the shear behavior of grain boundaries in Cu. Their results showed that the nanocavities could impede atom shuffling across the interfaces, preferentially facilitate the initiation of dislocation at cavities, and soften grain boundaries, resulting in a decrease of the yield strength. Recently, the growth of nanovoid in Al and Cu metals has been investigated using an improved QC method (HotQC), which considered the temperature effect [38]. It is known that surface orientation strongly affects the mechanical properties of the metals. However, the effect of cavities under different oriented surfaces of FCC metals on its mechanical properties has not been fully elucidated, especially for the (001) surface, which is generally believed to be the weakest surface of an FCC crystal [35]. Therefore, the cavity effect needs a more comprehensive study.

In this study, the QC method was used to investigate the effects of the depth, size, and shape of a single cavity on the onset plasticity of a lotus-type nanocavity containing a single crystal Al substrate nanoindentation on its (001) surface. We find that there is a critical depth of the cavity, over which the increase of contact stiffness and yield stress will tend to level off, and dislocations in the matrix are firstly observed to extend into the matrix after nucleated under the indenter edges and form a *y*-shaped structure. This could provide a guide to design nanoporous materials.

## 2. Quasicontinuum (QC) Model and Initialization

The QC method is a seamlessly coupled continuum and atomistic simulation method developed to simulate the static properties of crystalline solids across the discrete and continuum scales. It is established that in a crystalline solid under mechanical deformation, the majority of the solid undergoes a slowly varying deformation at the atomic scale, which can be well described by the continuum approximation and the discrete atomic effects become important only in the vicinity of defects, interfaces, and surfaces. Thus, there is no need to explicitly trace the status of every single atom in the crystal as in MD or lattice statics. During simulation, the atomistic domain would be automatically adjusted using a criterion based on local strain variation. More details of QC methodology can be found in Ref. [32].

For the simulation of aluminum, the Ercolessi and Adams potential [39] was used, as it agrees well with the basic experimental values [35]. The rectangular indenter is modeled in this simulation. Compared with the conical or spherical ones, the rectangular indenter is more conducive to avoiding producing geometrical necessary dislocation from the indenter to affect dislocation nucleation from the cavity and to obtaining accurate simulation results for the friction between the indenter and the material is negligible.

**Figure 1:**
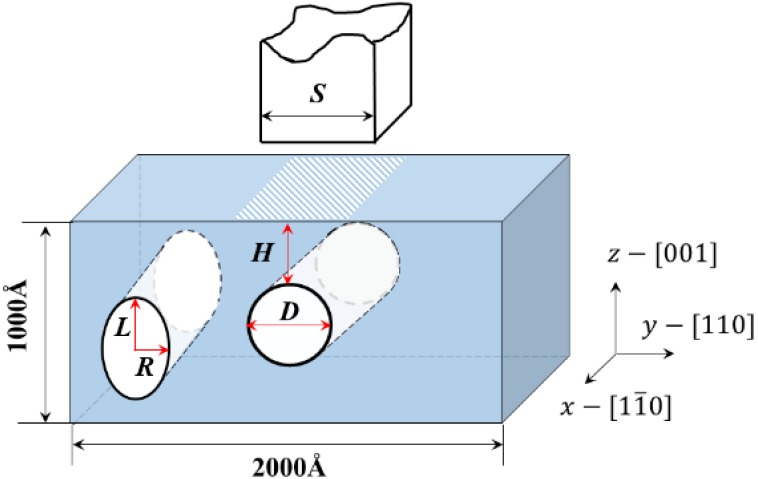
Schematic of the model of a single crystal Al with a lotus-type nanosized cavity during nanoindentation on the (001) surface, where *H*, *D*, *R*, and *L* are the depth between the top surface of the slab and the cavity top, the diameter of a circular cavity, the half-width and the half-height of an ellipsoidal cavity, respectively, and *S* is the width of the indenter.

Figure 1 shows the configuration of the simulation model. A pseudo two-dimensional model was used to simulate a thin slab with periodic boundary conditions applied in the out-of-plane direction (*x*-direction), resembling the “plane-strain” deformation in a continuum model. The dimensions of the single-crystal Al slab were 200 × 100 nm^2^ with an infinite thickness in the out-of-plane direction. The orientation of the matrix is $x-\left[1\bar{1}0\right],\;y-\left[110\right],\;z-\left[001\right]$, respectively. The matrix contained a single circular or elliptical cavity, where *H* was the depth between the cavity top and the top surface of the slab, *D* was the diameter of a circular cavity, *R* was the half-width of an ellipsoid cavity, *L* was the half-height of an ellipsoid cavity, and *S* was the indenter size.

A rectangular rigid indenter with a width of 80 Å was applied to the Al (001) surface in the [001] direction during the nanoindentation process. The width of 80 Å was chosen to be compatible with other published QC works [36,40], where the indenter size was 60 Å and 70 Å, respectively. Moreover, when the indenter width is larger than the diameter of cavity, it is convenient to observe the evolution of the microstructure and investigate the mechanism of the effect of cavity on the mechanical properties of the Al matrix. The width was set to be a variant only when the effect of the indenter width was investigated. The perfect-stick boundary condition was applied under the indenter in the [110] direction, where the top-layer atoms beneath the indenter could not move freely along this direction. Similar results were obtained when the friction-free boundary condition was applied. The atoms at the bottom of this slab were fixed. The system was firstly relaxed to eliminate internal stress before the load was applied. A depth-controlled indenter with an increment of 0.2 Å was used for the nanoindentation process. The temperature of the system was 0 K because of the QC limitation in the latest version (V. 1.4) of the software.

To obtain a stress–displacement curve during nanoindentation, we calculate the stress as the sum of the normal force under the indenter divided by the contact area and use the applied indenter displacement. In order to study the effect of the cavity on the stiffness of the material conveniently, we define the slope of stress–displacement curve in the elastic stage multiplied by contact area as the contact stiffness (*S_c_*) of the material. The von Mises strain is used throughout the present work, which has been previously demonstrated to effectively trace the dislocation slip-paths and interactions at the initial stages of plasticity during nanoindentation [35,41]. From color contrast, one can distinguish the magnitude of deformation. The von Mises strain is defined in Ref. [41] as

(1) $$\varepsilon^{\mathrm{Mises}}=\sqrt{\frac{{\left(\varepsilon_{xx}-\varepsilon_{yy}\right)}^{2}+{\left(\varepsilon_{yy}-\varepsilon_{zz}\right)}^{2}+{\left(\varepsilon_{zz}-\varepsilon_{xx}\right)}^{2}+6\left(\varepsilon_{xy}^{2}+\varepsilon_{yz}^{2}+\varepsilon_{xz}^{2}\right)}{2}}\;$$

where ε≡(12)log(C) is the logarithmic strain, defined in terms of the right Cauchy–Green deformation tensor $C=F^{T}F$, where *F* is the deformation gradient, defined as $F=1+u{\left(X\right)}^{\prime }$, derived from the displacement gradient $u{\left(X\right)}^{\prime }$, and $F^{T}$ is the transpose of matrix *F*. For deformations, the logarithmic strain can be approximately represented by the Lagrangian strain ε≈(12)(C−I), where *I* is the unit matrix.

## 3. Results and Discussion

### 3.1. The Stress–Displacement Curve and Dislocation Response of Onset Plasticity

**Figure 2:**
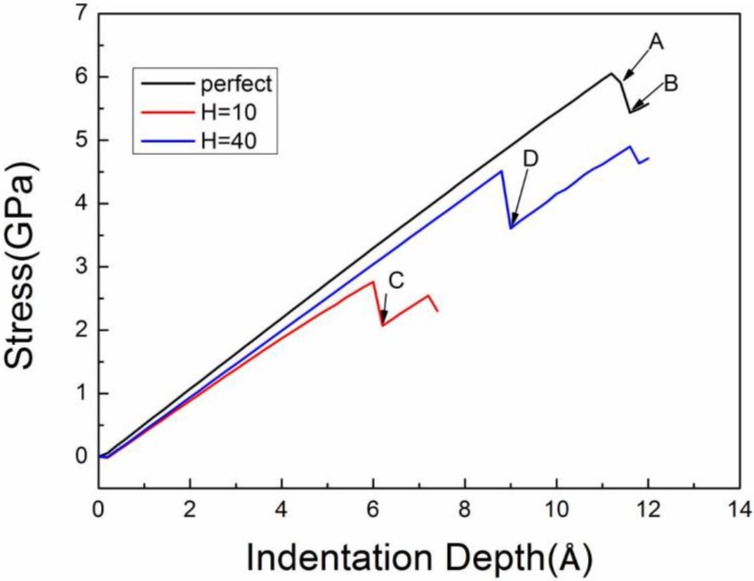
Three stress–displacement curves during nanoindentation of a perfect single crystal Al and two other Al matrixes containing a single circular cavity of *D* = 40 Å at the cavity depths of *H* = 10 Å and 40 Å.

In Figure 2, three stress–displacement curves during nanoindentation of Al matrixes with a circular cavity (*D* = 40 Å, *H* = 10 Å and *D* = 40 Å, *H*=40 Å) and a perfect single crystal Al are compared; the measurements were performed with an indenter size *S* = 80 Å. The stress initially decreases slightly because of the relaxation of the system to obtain a stable structure before the load is applied. Then, the stress–displacement curve shows a linear relationship corresponding to elastic deformation. The contact stiffness (*S_c_*) of the perfect Al without any cavity is the highest among the investigated matrixes. The *S_c_* of the Al matrix containing a single cavity increases with the cavity depth and gradually approaches that of the perfect Al.

The first force-drop event indicates the onset of plasticity, associated with dislocation nucleation, shown in Figure 2. The yield stress (*σ_y_*) is 6.1 GPa for the perfect Al matrix, and 2.8 GPa and 4.5 GPa for the Al matrixes with a single nanocavity (*D* = 40 Å, *H* = 10 Å) and (*D* = 40 Å, *H* = 40 Å), respectively. Moreover, the *σ_y_* of the matrixes with a single cavity (*D* = 40 Å, *H* = 80 Å) is approximately 6.0 GPa, which is close to the perfect one. Yu et al. [36] also found that the yield stress of a perfect thin film served as the upper limit of that with a cavity in the study of cavity effect on the mechanical properties of Cu film during nanoindentation on a (110) surface.

**Figure 3:**
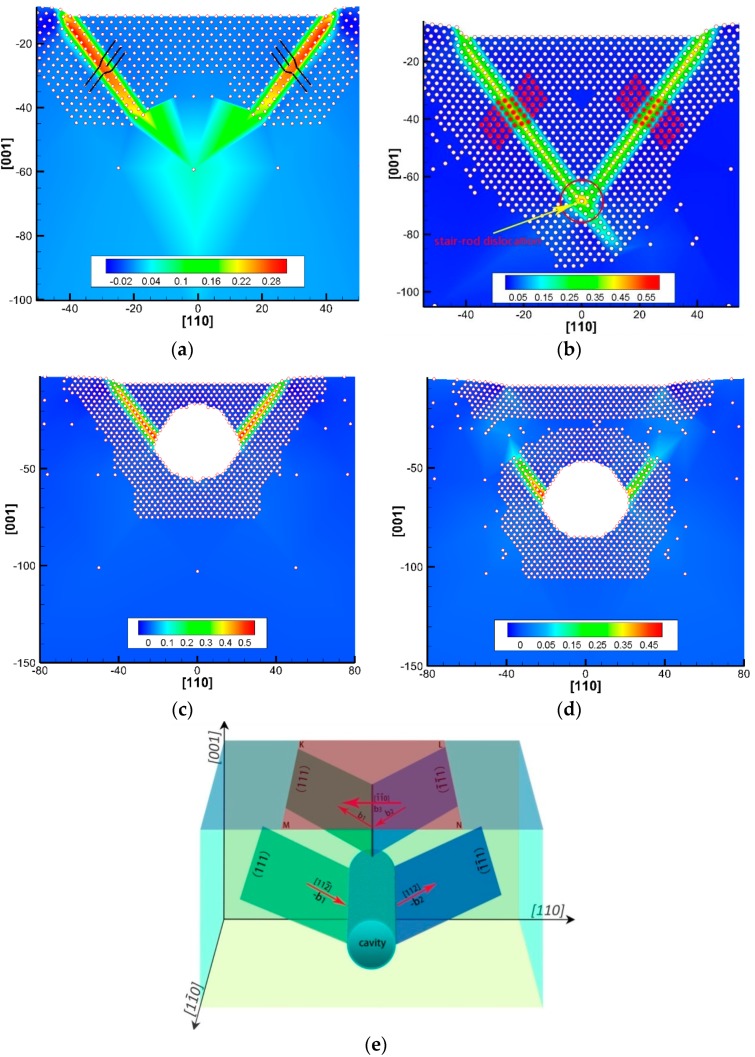
The dislocation structure at different yield point in Figure 2, (**a**) the ‘A’ point, (**b**) the ‘B’ point, (**c**) the ‘C’ point and (**d**) the ‘D’ point, and (**e**) schematic of two 1/6 <112> Shockley partial dislocations with Burgers vector of b_1_ and b_2_ on the (111) and $\left(\bar{1}\bar{1}1\right)$ plane, respectively, and a stair-rod dislocation formed by the reaction of two partials, where the ‘KLMN’ region is the punching zone under the indenter.

To further understand the yielding mechanism, we plotted the von Mises strain (defined in Equation (1)) contour at the yield points of A, B, C, and D in Figure 2, shown in Figure 3a–d. The yield points are observed to correspond to dislocation nucleation; the dislocation types are 1/6 <112> Shockley partial and 1/3 <110> stair-rod dislocations, as confirmed in our previous work [35]. For the perfect single crystal Al, two Shockley partials nucleate under the indenter edges (at yield point A), with a Burgers vector of b_1_ =$\;\frac{1}{6}a\left[\bar{1}\bar{1}2\right]$ on the (111) plane and b_2_ =$\;\frac{1}{6}a\left[\bar{1}\bar{1}\bar{2}\right]$ on the $\left(\bar{1}\bar{1}1\right)$ plane, shown in Figure 3a,e. Further yielding leads to the formation of a $\frac{1}{3}a\left[\bar{1}\bar{1}0\right]$ stair-rod dislocation with the reaction of $\frac{1}{6}a\left[\bar{1}\bar{1}2\right]+\frac{1}{6}a\left[\bar{1}\bar{1}\bar{2}]\to \frac{1}{3}a[\bar{1}\bar{1}0\right]$ at their intersect point [42], followed by a two-layer thickness twin generation due to the nucleation and slip of a new set of Shockley partials on the adjacent atomic layer, as shown in Figure 3b. However, when the cavity is presented in the Al matrix, it cannot only substantially reduce the yield stress but also affect the dislocation structure. Two partials nucleation from the inner side of the cavity surface (*D* = 40 Å) is observed in Figure 3c,d. Instead of the formation of a stair-rod dislocation in the perfect Al, these dislocations are escaped from the cavity surface. The deformation twins are also generated. Our results support the observations in other works, where the stress intensity at the cavity were twice as that of an area far from the cavity [30] and the local stress concentration can facilitate dislocation nucleation [29].

### 3.2. The Effect of the Depth of the Single Circular Cavity on Onset Plasticity

**Figure 4:**
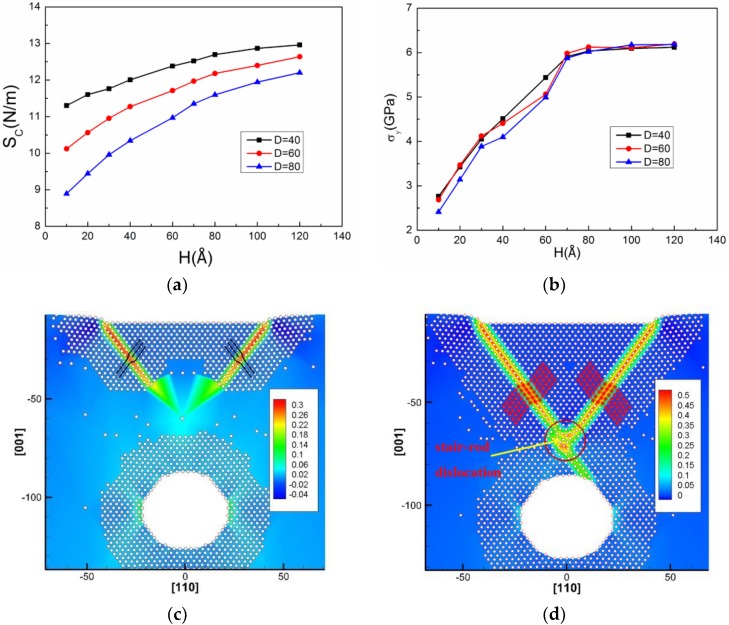
(**a**) Contact stiffness (*S_c_*) and (**b**) yield stress (*σ_y_*) of the Al with a circular cavity at different depths (*H*), respectively. The dislocation morphology in the matrix with the cavity (*H* = 80 Å, *D* = 40 Å, *S* = 80 Å) (**c**) at the yield point and (**d**) further yielding.

In this section, we discuss the effect of the cavity depth on contact stiffness and yield stress. The variant depth (*H*) of the circular cavity is selected (note: the exact value of *H* should be the times of Al lattice constant). The values of contact stiffness and yield stress during nanoindentation as functions of cavity depth are plotted in Figure 4, along with their associated dislocation responses at initial yielding.

The results show that firstly, the *S_c_* and *σ_y_* of the Al matrix gradually increase with increasing depth *H* and then tend to saturate after a certain depth in Figure 4a,b, which indicates that a critical depth (*H*_0_) exists. Similar observation about modulus was also reported by Yang et al. [31] who used MD simulations to investigate the mechanical behavior of a single-crystalline Cu plate with nanocavities under uniaxial tension, where the elastic modulus linearly decreased with increasing *D* and nonlinearly increased with the aspect ratio (*AR*). Yu et al. [36] used the QC method to investigate the effects of the shape and position of the cavity on the mechanical properties of the Cu film during nanoindentation on a (110) surface. They indicated that the increase of nanocavity depth leads to yield stress approaching that of the perfect film.

The *H*_0_ is around 80 Å in the matrix with the circular cavity (*D* = 40 Å, *S* = 80 Å). Figure 4c shows that the first yield point corresponds to two partial dislocations of the nucleation under the indentation edges, rather than from the inner side of the cavity. For further yielding, the nucleated dislocations extend into the matrix without touching the cavity, which then form a *y*-shaped structure, as shown in Figure 4d. The *y*-shaped dislocation structure was explained by Jin et al. [35], who studied the onset plasticity of an Al (001) surface under a rectangular indenter. They indicated that the *y*-shaped structure was formed by a deformation twin in one side and a partial dislocation in the other side, intersected by a stair-rod dislocation.

### 3.3. The Effect of the Diameter of the Single Circular Cavity on Onset Plasticity

**Figure 5:**
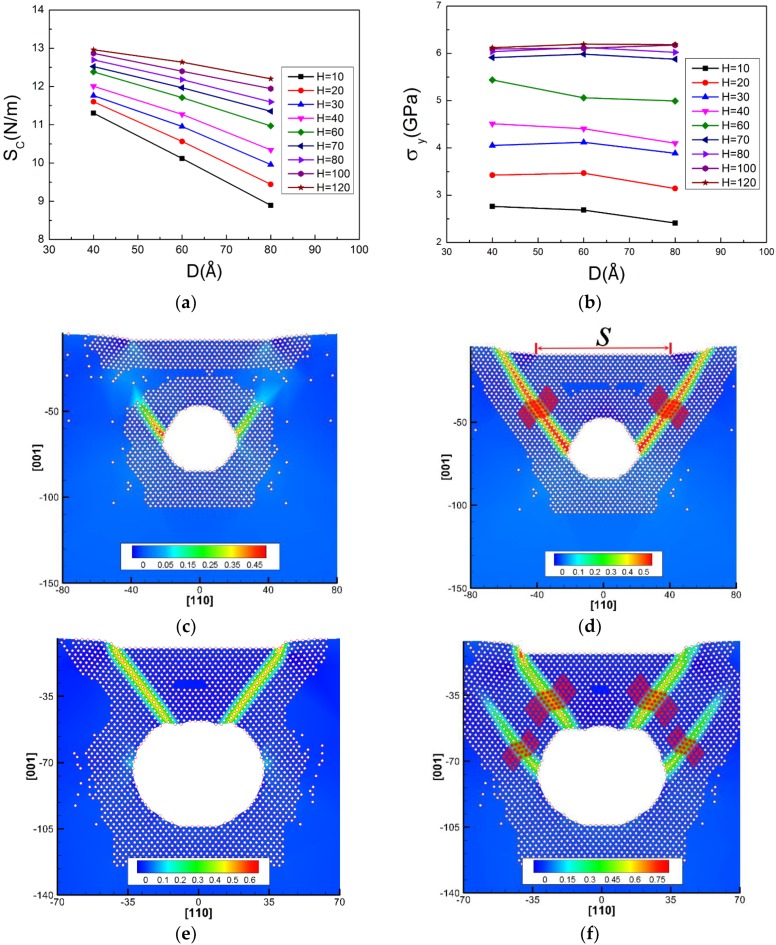

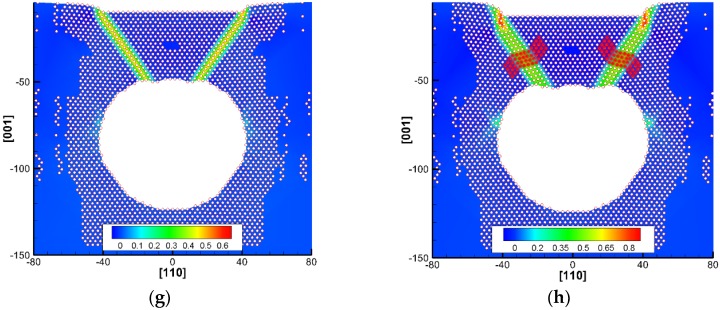
(**a**) Contact stiffness (*S_c_*) and (**b**) yield stress (*σ_y_*) of Al containing a circular cavity with different diameter (*D*) during nanoindentation. The dislocation morphology in the matrix (**c**,**e**,**g**) at the yield point and (**d**,**f**,**h**) further yielding with the cavity (*D* = 40 Å, *H* = 40 Å, *S* = 80 Å), (*D* = 60 Å, *H* = 40 Å, *S* = 80 Å) and (*D* = 80 Å, *H* = 40 Å, *S* = 80 Å).

The effect of the diameter (*D*) of the circular cavity on the *S_c_* and *σ_y_* of the Al matrix is discussed in this section. The *S_c_* decreases monotonically with increasing *D* (Figure 5a), whereas *σ_y_* is only slightly affected by *D* (Figure 5b). In Zhou et al.’s work [43], where the surface effect on Young’s modulus of Cu nanoplates was investigated by MD simulation, it was found that Young’s modulus of the nanoplate was a function of its thickness and greatly different with that of bulk Cu, especially when the nanoplate thickness was less than 40 atomic layers. 

It is also found that the site for dislocation nucleation at the yield point is different when *D* is changing from 40 to 80 Å. In Figure 5c,d, the dislocation nucleates at the inner side of the cavity surface when the *D* is equal to 40 Å, which is also confirmed by dislocation morphology at further yielding. Then these nucleated dislocations slip and eventually escape from the Al surface. Conversely, the sites for dislocation nucleation change from the cavity surface to the indentation edges in Figure 5g when the *D* is 80 Å, which can also be proved by dislocation structure in Figure 5h. Then these nucleated dislocations slip and eventually escape from the cavity surface. When we study the dislocation structure in the matrix containing the cavity with *D* = 60 Å in Figure 5e,f, it shows that the dislocation can possibly nucleate from either of the two-type sites discussed above. Therefore, these results reveal that there are two types of nucleation sites in the cases with the cavity diameter ranging from 40 to 80 Å, and the transition diameter is about 60 Å. However, when the cavity depth is over the critical depth, the nucleated sites are only under the indenter edges in all cases. Moreover, regardless of the depth effect of the cavity, since the type of the nucleated partial dislocation at initial yielding is the same, the corresponding yield stress of the matrix should be very close.

### 3.4. The Effect of the Aspect Ratio of Single Elliptical Cavity on Onset Plasticity

**Figure 6:**
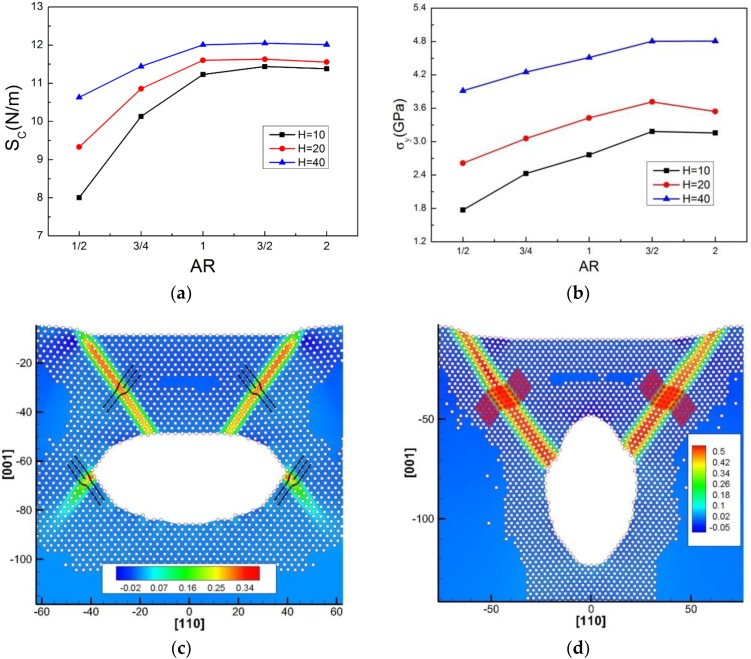
(**a**) Contact stiffness (*S_c_*) and (**b**) yield stress (*σ_y_*) of Al containing an elliptical cavity with different aspect ratios *AR* (*AR* = *L*/*R*) during nanoindentation. The dislocation morphology of the matrix at the yield point for the cavity cases of (**c**) (*AR* = 1/2, *H* = 40 Å, *R* = 40 Å, *S* = 80 Å) and (**d**) (*AR* = 2, *H* = 40 Å, *R* = 20 Å, *S* = 80 Å).

As the stress concentration near the cavity strongly depends on its shape [27], the effect of the aspect ratio (*AR*) of the elliptical cavity on the *S_c_* and *σ_y_* of the Al is discussed in this section. Figure 6a,b show that the *S_c_* and *σ_y_* increase with the aspect ratio (*AR* = *L*/*R*, where *L* and *R* are the half-height and half-width of an elliptical cavity, respectively), while they are less affected when the *AR* is over 1. In the work [31], it was also found that the elastic modulus increased with the *AR* of the cavity. Our results indicate that the effect of the elliptical cavity on mechanical properties of the Al matrix is more significant when the cavity is oblate.

In Figure 6c, the dislocation evolution at yielding is that two partial dislocations appear at the sharp corners of the cavity, then two partials nucleate under the indenter edges, slip into the matrix and escape from the cavity surface eventually. In Figure 6d, the dislocation evolution at yielding is described as follows: (1) two partial dislocations nucleate at the inner side of the cavity surface, move upward and then escape from the Al surface; (2) other partials nucleate next to the former partials until they escape and (3) the deformation twins are formed eventually. A comparison of dislocation morphology in the matrix with the oblate (*AR* = 1/2) and prolate (*AR* = 2) cavities in Figure 6c,d reveals that the corners of the oblate cavity may also facilitate dislocation nucleation, where these corners are commonly known as a high stress concentration area for cracking. However, it is noted that these dislocations nucleated from the cavity corners are very difficult to slip into the matrix, as shown in Figure 6c.

### 3.5. The Effect of Indenter Size on the Critical Depth

In Section 3.2, where the effect of the cavity depth is discussed, a critical depth (*H*_0_) is observed, which is identified on the basis of (1) the saturation of *S_c_* and *σ_y_* and (2) the first observation where the partial dislocations extend into the matrix after being nucleated under the indenter edges and then form a *y*-shaped dislocation structure. Moreover, it was also found that the *H*_0_ differed with the size of the indenter. Therefore, the effect of indenter size *S* on the *H*_0_ will be studied in this section.

**Figure 7:**
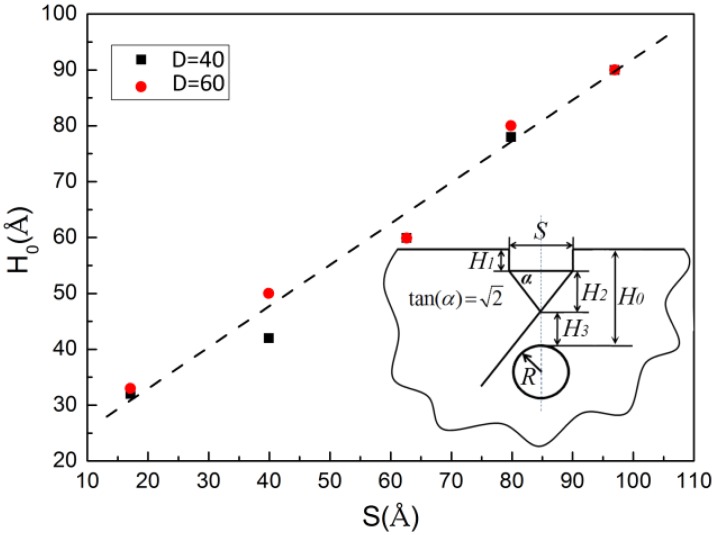
The relationship between the critical depth (*H*_0_) and indenter size (*S*).

To investigate the relationship between *H*_0_ and *S*, we selected a circular cavity with two diameters of 40 Å and 60 Å and varied the indenter size from 17 Å to 97 Å. Surprisingly, it is found that *H*_0_ and *S* exhibit an approximately linear relationship fitted with a function of *H*_0_ = *H*_1_ + *H*_2_ + *H*_3_ = 0.74*S* + 17.82, where *H*_1_ is the indentation depth at initial yielding, *H*_2_ is the height of the *y*-shaped structure and *H*_3_ is the distance between the cavity top and the intersection point of the *y*-shaped one, as shown in Figure 7. This relationship is likely related to dislocation nucleation sites and the geometric relationship between the slip system and the *S*, as inset in Figure 7, which satisfies a *y*-shaped structure. The slope is 0.74 rather than $\sqrt{2}/2$, which could be a result of the effect of the *H*_1_. The value of 17.82 is suggested to be *H*_3_ and is about a length of five to eight atomic layers, which might be a minimum space to accommodate a dislocation core of 1/3 <110> stair-rod dislocation. Additionally, it is also noted that the angle *α* would be slightly changed due to the rotation of the slip plane during deformation.

### 3.6. Surface Orientation Effect

Due to the non-isotropic mechanical property in an Al single crystal in addition to our nanoscale-size of model, our results are affected by the surface orientation. To what extent does such an effect deserve further study? Here we discuss the orientation effect on the onset plasticity of a single crystal Al containing nanocavity. The mechanism of onset plasticity has been investigated for the (100), $\left(\bar{1}10\right)$ and the (111) surfaces of a perfect single crystal Al matrix in our previous work [35].

**Figure 8:**
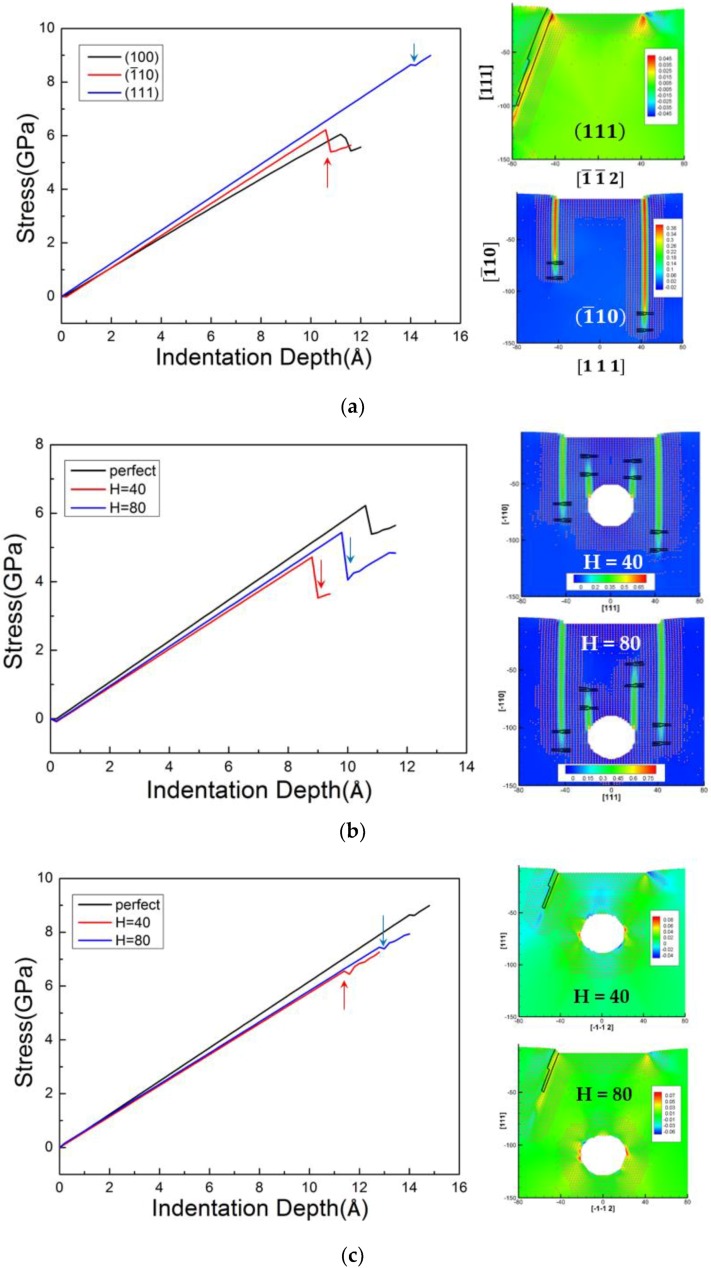
Stress–displacement curves of nanoindentation (**a**) on the (001), $\left(\bar{1}10\right)$ and (111) surfaces of a perfect Al single crystal, (**b**) on the $\left(\bar{1}10\right)$ and (**c**) on the (111) surface of a perfect Al single crystal and two matrixes with a nanocavity (*D* = 40 Å, *H* = 40 Å) and (*D* = 40 Å, *H* = 80 Å) respectively. The side insets are dislocation morphology at the initial yielding point.

Compared with the (001) surface, the magnitude of the initial load-drop and the corresponding dislocation morphology are different, as shown in Figure 8a. The yield stress (*σ_y_*) of the initial load-drop on the (001) surface is 6.1 GPa, smaller than that on the (110) surface (6.2 GPa), and the (111) surface (8.6 GPa), which proves that the (001) surface produces a relatively low mechanical resistance in the Al single crystal. The average yield stress of a perfect Al single crystal during nanoindentation on three different surfaces is about 7.0 GPa. Moreover, on the $\left(\bar{1}10\right)$ surface, the initial load-drop is sharp, and the dislocations are of a dissociated edge type: each consisted of two 1/6 <112> Shockley partials with a stacking fault in between. On the (111) surface, the initial load-drop is very small and the 90° Shockley partial beneath the indenter is $1/6\left[\bar{1}\bar{1}\bar{2}\right]$ in the $\left(\bar{1}\bar{1}1\right)$ plane initiated from the left-hand corner of the indenter.

For the Al matrix with a nanocavity (*D* = 40 Å, *H* = 40 Å) as shown in Figure 8b,c, the *σ_y_* is about 4.7 GPa for the $\left(\bar{1}10\right)$ surface and 6.6 GPa for the (111) surface, respectively, and the average yield stress from three surfaces is 5.3 GPa. While, the *σ_y_* of the matrix with the cavity (*D* = 40 Å, *H* = 80 Å) becomes 5.4 GPa and 7.5 GPa, respectively, and the average stress is 6.3 GPa, which is closer to that of the perfect Al. The dislocation morphology shows that the nucleation site can be beneath the indenter edges and the inner surface of the cavity for the $\left(\bar{1}10\right)$ surface, while it is only from the indenter corner for the (111) surface, which is the same as the perfect Al crystal.

## 4. Conclusions

We have investigated the effects of the depth, size, and shape of a single nanocavity on the onset plasticity of a single crystal Al during nanoindentation on a (001) surface using the quasicontinuum method. We find that the existence of nanocavities in the Al matrix can affect the contact stiffness and yield stress of the matrix during nanoindentation, especially when the cavity is near the Al surface. The contact stiffness and yield stress gradually increase with increasing depth of the circular cavity (*H*) (i.e., the distance between the surface and the cavity top). A critical depth (*H*_0_) exists, which can be identified on the basis of the first observation of a y-shape dislocation structure. The increasing contact stiffness and yield stress will tend to be saturated when *H* is greater than *H*_0_. The contact stiffness of the matrix almost linearly decreases with the increasing cavity diameter (*D*), whereas the yield stress is only slightly affected by the *D*. The contact stiffness and yield stress of the Al matrix increase significantly with the aspect ratio *AR* of the elliptical cavity, while they are less affected when the *AR* is over 1. In the case of cavities with the same radius, the critical depths (*H*_0_) from different-sized indenters exhibit an approximately linear relationship with the indenter size.
